# Effect of Transcutaneous Upper Eyelid Blepharoplasty on Blink Parameters and Lipid Layer Thickness

**DOI:** 10.3389/fmed.2021.732041

**Published:** 2021-11-22

**Authors:** Siyi Zhang, Yan Yan, Yang Lu, Yixiong Zhou, Yao Fu

**Affiliations:** ^1^Department of Ophthalmology, Shanghai Ninth People's Hospital, Shanghai JiaoTong University School of Medicine, Shanghai, China; ^2^Shanghai Key Laboratory of Orbital Diseases and Ocular Oncology, Shanghai, China

**Keywords:** upper blepharoplasty, blink, lipid layer thickness (LLT), dry eye, noninvasive tear film breakup time (NITBUT)

## Abstract

**Purpose:** This study aimed to investigate the effect of transcutaneous upper eyelid blepharoplasty on lipid layer thickness (LLT) and blink parameters in young women during the early postoperative period.

**Methods:** This prospective study included 110 eyes of 55 young female patients (age range, 19–31 years) who underwent transcutaneous upper eyelid blepharoplasty. The LLT and blink parameters measured using a LipiView interferometer were recorded before the surgery and 1 week and 1 month after the surgery. Ocular Surface Disease Index (OSDI) score, noninvasive tear film breakup time (NITBUT), and palpebral fissure height (PFH) measurements were also performed at each time point.

**Results:** The number of blinks significantly decreased (*P* < 0.001), and the number of partial blinks and partial blink rate (PBR) significantly increased 1 week after the surgery (*P* = 0.002 and *P* < 0.001); they all returned to baseline in 1 month. The LLT and OSDI score increased significantly 1 week and 1 month after the surgery (*P* < 0.001 and *P* < 0.001). A significant increase in the NITBUT and a significant decrease in the PFH were observed 1 week after the surgery (*P* < 0.001 and *P* < 0.001), and the values returned to baseline 1 month after the surgery. No clinical correlation was found between blink parameters and ocular surface parameters.

**Conclusions:** Transcutaneous upper eyelid blepharoplasty led to a change in blink parameters and ocular surface parameters during the early postoperative period. However, this influence was temporary, and the transitory change in blink parameters had no effects on the ocular surface environment.

## Introduction

Some Asians have a single upper eyelid or upper eyelid hypertrophy, which makes their eyes appear small and droopy, owing to the difference in fat distribution in the orbital septum and upper eyelid anatomical structures between Asians and the white race (1). Many Asians, especially young women, choose to undergo upper blepharoplasty to have a pair of attractive double eyelids. However, this surgical procedure is associated with many complications (2, 3), and dry eye disease (DED) is one of the most common complications (4). A retrospective study of 892 patients indicated that dry eye symptoms following blepharoplasty were reported in 26.5% of patients (4). Yan et al. measured subjective and objective parameters of dry eye in patients who underwent upper blepharoplasty and found that upper eyelid blepharoplasty might temporarily affect tear film dynamics and aggravate dry eye symptoms in young women. However, these changes generally disappear 3 months after the surgery (5). Zloto et al. (6) also confirmed no significant differences between the postoperative and preoperative objective and subjective dry eye tests in patients with blepharoplasty 3 months after the surgery.

However, DED is a multifactorial ocular surface disease characterized by a loss of homeostasis of the tear film (7), and a stable preocular tear film is a hallmark of ocular health (8). The tear film consists of a mucin layer, an aqueous layer, and a lipid layer. The lipid layer is important in preventing the loss of aqueous layer through overspill and evaporation (8–11). Upper blepharoplasty involved surgical removal of the skin, orbicularis oculi muscle, and (or) orbital fat, and might lead to the formation of scar and injury to the innervation, resulting in incomplete blink and decreased blink rate (3, 12). The fullness of the blink affects the stability of the tear film and its distribution in the inter-blink period (13). Moreover, the mechanical action of the lid muscles during the blink contributes to the delivery of meibomian oil (9). Jie et al. (14) indicated that partial blink would contribute to meibomian gland obstruction and subsequent loss of tear film homeostasis. Therefore, an abnormal eyeblink pattern was considered as a part of DED pathogenesis. Previously, few studies focused on the specific influence of upper blepharoplasty on eyeblink and lipid layer (15). Whether the postoperative dry eye symptoms have a correlation with the change in blink parameters and lipid layer is unclear. Thus, the lipid layer thickness (LLT) and blink parameters before and after the surgery need to be evaluated, and the specific influence of transcutaneous upper blepharoplasty on the LLT and blink parameters needs to be confirmed.

Eyeblink is a fast eyelid movement that closes and opens the palpebral fissure (13, 16, 17); it is difficult to measure and analyze blink parameters. Recently, some new devices are being used to analyze eyeblink patterns. One of these is a LipiView interferometer (TearScience, NC, USA), which provides clinicians with the LLT of the tear film and partial blink rate (PBR) (14, 18, 19).

Therefore, this prospective study was designed to observe the effect of transcutaneous upper eyelid blepharoplasty on the LLT, blink parameters, and tear film stability, and also evaluate the relationship between blink parameters, LLT, and tear film stability using the LipiView interferometer.

## Methods

This single-institution prospective, observational clinical study was approved by the ethics committee of the Shanghai Ninth People's Hospital. The study began on January 1, 2019, and lasted for 10 months. It was conducted in accordance with the 1964 Helsinki declaration and its later amendments or comparable ethical standards. Prior written informed consent was obtained from all patients after receiving a detailed explanation of the study protocols and possible consequences associated with participation.

### Participants

A total of 55 female patients (110 eyes) referred to the Department of Ophthalmology of Shanghai Ninth People's Hospital, who underwent upper eyelid blepharoplasty, were included in the study.

Patients with a history of ophthalmic surgeries (intraocular surgeries, eyelid surgeries, etc.); previous dry eye disease; systemic diseases as thyroid eye disease, disorders of the eyelids, and other ophthalmic diseases that required priority treatment; glaucoma; incomplete medical patient records, and age <18 years or >35 years were excluded from the study.

### Surgical Procedures

The surgeries were performed by a single surgeon (Y.F.) using the same techniques. The incision line was marked about 6–10 mm above the upper lid. The orbicularis oculi muscle was removed inferior to the incision within 2–3 mm. Then, the septum was incised to expose and remove the fat tissue over the top of the tarsus. The incision was closed using a 6–0 silk suture through the skin and aponeurosis.

### Clinical Evaluation

All patients were scheduled for examinations before the surgery and 1 week and 1 month after the surgery. At each scheduled time point, subjective symptoms were evaluated using the ocular surface disease index (OSDI) questionnaire, and then clinical examinations were performed in the following order: lipid layer thickness (LLT) measurement, blink parameter analysis, noninvasive tear breakup time (NITBUT), and palpebral fissure height (PFH). All the clinical examinations were performed by a single clinician (SZ) at the same room location at each visit.

The LLT and blink parameters were analyzed with the LipiView interferometer. The patients positioned their eyes in front of an illumination source and were asked to blink freely, and a 20-s video was captured and recorded. The interferometric color unit value reflected the local LLT with 1 color unit equivalent to 1 nm of lipid layer thickness. The number of blinks and partial blinks were recorded, and PBR was calculated as the rate between the two.

The NITBUT was recorded using the Keratograph 5M (Oculus, Optikgerate, Germany). The examination was carried out and repeated three times under the condition of natural eye opening, and the average value was recorded.

The PFH was measured using an imaging system designed based on a convolutional neural network. The images of the eyes were documented at each time point.

### Statistical Analysis

Statistical analysis was performed with SPSS for Windows version 25.0 (SPSS Inc., IL, USA). Descriptive statistics were expressed as mean ± standard error (SE). Data were examined for normality using the Shapiro–Wilk test. A generalized estimation equation (GEE) was used to compare the variables of interest among different visits. The working correlation for each GEE model was selected using the corrected quasi-likelihood under the independence model criterion. The Spearman correlation coefficient (*r*) was calculated to assess the relationship between blink parameters, LLT, NITBUT, and OSDI value. All tests were two-tailed, and a *P* < 0.05 was considered significant.

## Results

A total of 110 eyes of 55 female patients were identified. The mean age of the participants was 25.18 ± 2.99 (mean ± standard deviation) years (range, 19–31 years). The ocular findings based on routine ancillary tests performed in the cornea clinic are shown in Table 1.

**Table 1:** Comparison of preoperative and postoperative ocular surface parameters (**P < 0.001).

**Ocular surface parameters**	**Before the surgery**	**After the surgery**		***P*** **value**	
		**1 week**	**1 month**	**1 week after the surgery vs. before the surgery**	**1 month after the surgery vs. before the surgery**
OSDI	6.58 ± 0.36	8.89 ± 0.41	11.95 ± 0.55	<0.001**	<0.001**
LLT_av_, nm	76.74 ± 2.51	89.44 ± 1.75	90.85 ± 1.72	<0.001**	<0.001**
NITBUT_av_, s	8.91 ± 0.40	11.66 ± 0.36	8.72 ± 0.35	<0.001**	0.17
Number of blinks	7.40 ± 0.18	6.36 ± 0.17	7.43 ± 0.20	<0.001**	0.73
Number of partial blinks	4.22 ± 0.34	5.12 ± 0.24	4.29 ± 0.38	0.002**	0.57
PBR	0.58 ± 0.05	0.81 ± 0.03	0.58 ± 0.04	<0.001**	0.80
PFH, mm	9.45 ± 0.12	8.52 ± 0.13	9.31 ± 0.11	<0.001**	0.08

**Figure 1:**
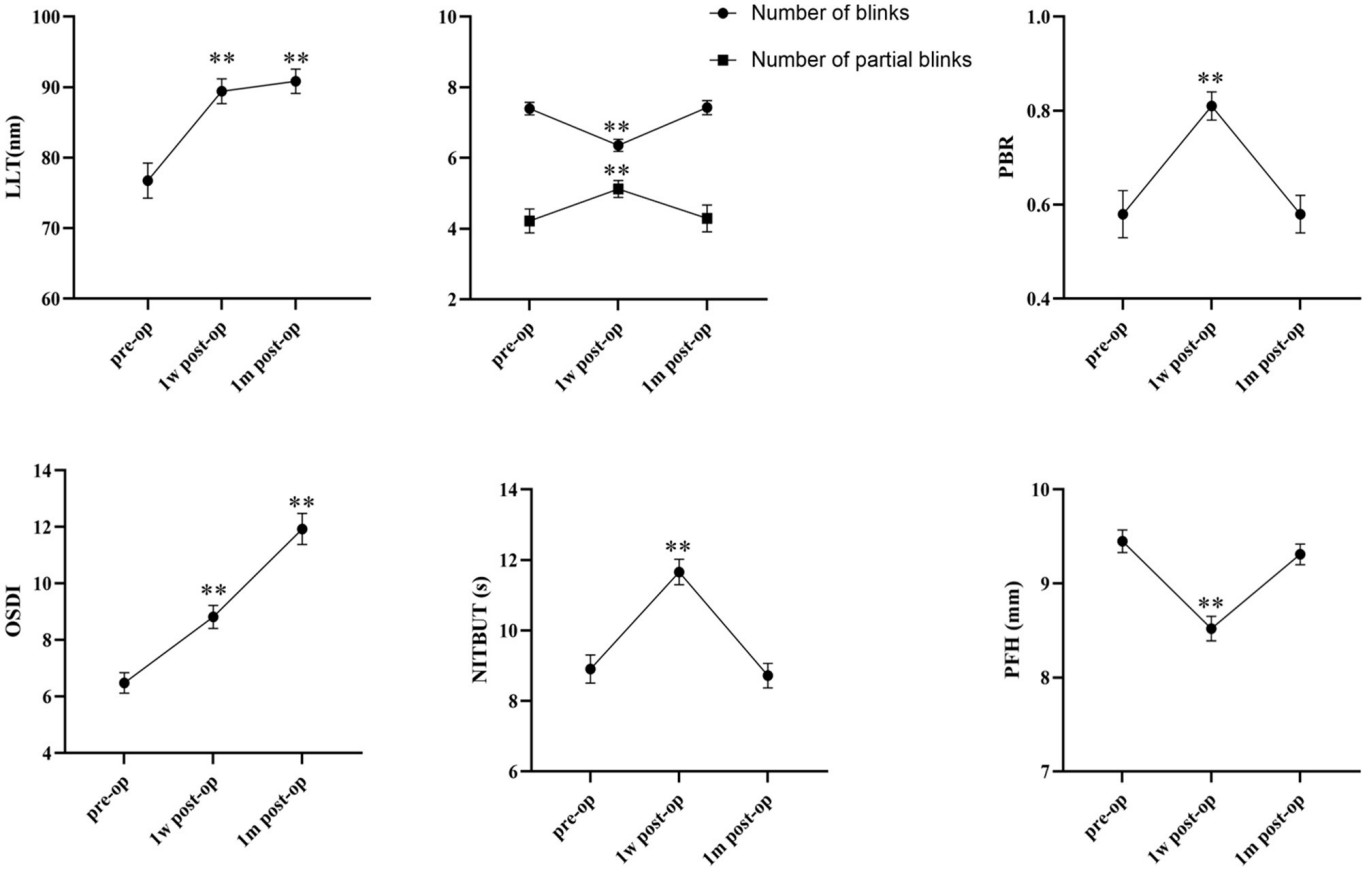
Graph depicting mean and standard error (SE) for OSDI, LLT, NITBUT, PFH, number of blinks, number of partial blinks, and PBR before and after upper blepharoplasty (**P < 0.001) (pre-op, preoperative; post-op, postoperative; 1w, 1 week; 1m, 1 month).

A significant decrease was observed in number of blinks and PFH 1 week after the surgery; these values returned to baseline 1 month after the surgery (number of blinks: before the surgery, 7.40 ± 0.18; 1 week after the surgery, 6.36 ± 0.17, *P* < 0.001; 1 month after the surgery, 7.43 ± 0.20, *P* = 0.73; PFH: before the surgery, 9.45 ± 0.12; 1 week after the surgery, 8.52 ± 0.13, *P* < 0.001; 1 month after the surgery, 9.31 ± 0.11, *P* = 0.08). A significant increase in the number of partial blinks and PBR was observed 1 week after the surgery, and the values 1 month after the surgery had no significant differences compared with preoperative values (number of partial blinks: before the surgery, 4.22 ± 0.34; 1 week after the surgery, 5.12 ± 0.24, *P* = 0.002; 1 month after the surgery, 4.29 ± 0.38, *P* = 0.57; PBR: before the surgery, 0.58 ± 0.05; 1 week after the surgery, 0.81 ± 0.03, *P* < 0.001; 1 month after the surgery, 0.58 ± 0.04, *P* = 0.80). The OSDI value and LLT significantly increased 1 week after the surgery and increased further 1 month after the surgery (OSDI: before the surgery, 6.48 ± 0.36; 1 week after the surgery, 8.82 ± 0.41, *P* < 0.001; 1 month after the surgery, 11.93 ± 0.55, *P* < 0.001; LLT: before the surgery, 76.74 ± 2.51; 1 week after the surgery, 89.44 ± 1.75, *P* < 0.001; 1 month after the surgery, 90.85 ± 1.72, *P* < 0.001). The mean NITBUT increased significantly and returned to preoperative levels 1 week after the surgery (before the surgery, 8.91 ± 0.40; 1 week after the surgery, 11.66 ± 0.36, *P* < 0.001; 1 month after the surgery, 8.72 ± 0.35, *P* = 0.17; Figure 1).

The number of blinks had a statistically significant correlation with NITBUT and LLT (*r* = −0.13, *P* = 0.02; *r* = −0.13, *P* = 0.02). The PFH correlated significantly with the NITBUT (*r* = −0.12, *P* = 0.03); but the coefficients of correlation were less than 0.2 and had no clinical significance. No correlation was found between the other parameters (Table 2).

**Table 2:** Correlation between blink parameters and ocular surface parameters (*P < 0.05).

		**OSDI**	**NITBUT**	**LLT**
Number of blinks	*r*	0.03	−0.13	−0.13
	*P*	0.59	0.02*	0.02*
Number of partial blinks	*r*	0.02	0.04	−0.02
	*P*	0.72	0.46	0.74
PBR	*r*	0.03	0.09	0.05
	*P*	0.64	0.11	0.40
PFH	*r*	−0.08	−0.12	−0.08
	*P*	0.13	0.03*	0.13

## Discussion

DED is caused by a variety of iatrogenic interventions, and one of the most emblematic situations is DED caused by surgical procedures (20). The increasing number of patients looking for cosmetic procedures has drawn more attention to DED caused or worsened by blepharoplasty (21). Studies indicated that the prevalence of DED after blepharoplasty ranged from 0 to 26.5% (3, 4, 22). Previous studies investigated the changes in tear film parameters after upper blepharoplasty (6, 23), but only a few of them paid attention to the changes in lipid layer and eyeblink parameters. Therefore, this study was performed to observe the specific influence of transcutaneous upper blepharoplasty on LLT, blink parameters, and tear film stability in young Asian women during the early postoperative period, using the LipiView interferometer and Keratograph 5M.

The results showed that blink patterns changed 1 week after the surgery and returned to baseline in 1 month. The number of blinks decreased while the number of partial blinks and PBR increased 1 week after the surgery. Eyeblink is a complex process influenced by many factors, such as ocular surface damage and exposure, corneal and lid margin sensitivity, muscular fatigue and tension, and so on (16). The active forces that produce the movement of the upper eyelid during a blink are generated only by the orbicularis oculi muscle and levator palpebral superior muscle. The transcutaneous upper blepharoplasty performed in the present study involved the removal of the orbicularis oculi muscle (2–3 mm) and periorbital fat. The surgical procedure caused tissue trauma, which led to inflammatory responses and postoperative periorbital swelling. During the early postoperative period, patients blinked less and incompletely due to the swelling and incision pain. However, this phenomenon was temporary. Patients were used to the condition, and the incision pain was greatly eased 1 month after the surgery. Hence, the blink patterns returned to baseline, indicating that the limited excision of the orbicularis oculi muscle did not affect blink patterns in young patients.

In this study, the postoperative LLT significantly increased compared with the preoperative value. The driving forces leading to meibum secretion onto the lid margin and tear film are responsible for the mechanical muscular action by muscle fibers of the pretarsal orbicularis muscle, located on the outside of the tarsus, and of the marginal muscle of Riolan, which encircles the terminal part of the meibomian gland (9). The transcutaneous blepharoplasty removed part of the orbicularis muscle, but did not involve the muscle of Riolan, thus retaining the maximum driving force on the meibomian glands. The meibum is secreted by the meibomian gland through the thickness of the lids due to pressure on the glands (10). The upper eyelid is thickened due to surgical damage, and the postoperative swollen eyelid contributes to the increased pressure on the meibomian glands. The PFH decreased during the early postoperative period, indicating a reduction in the ocular surface area. Therefore, the LLT increased 1 week and 1 month after the surgery due to increased meibum secretion and decreased ocular surface area.

Previous experiments have shown that tear film breakup occurs mainly as a result of evaporation from the tear film (8). The lipid layer is of great importance for the stabilization of the air/aqueous tear interface of the tear film (11). Thus, the NITBUT increased 1 week after the surgery and returned to baseline 1 month after the surgery, indicating that the effect of blepharoplasty on the tear film breakup time was temporary.

However, despite the increased LLT and NITBUT, the results showed that OSDI values increased 1 week and 1 month after the surgery, demonstrating the worsening of the subjective symptoms of the patients. Although the patients' objective signs improved, they still complained of suffering from more severe dry eye symptoms. The main complaints of the patient were the foreign body sensation and irritation. The decreased number of blinks and increased PBR would aggravate the foreign body sensation and irritation, and led to increase of OSDI values (16). And it was assumed that postoperative inflammation and incision pain might aggravate the subjective feeling.

In addition, no correlation was observed between blink parameters and ocular surface parameters. Previous studies showed that partial blink correlated with ocular surface parameters (14, 19). However, these studies were performed on middle-aged and elderly patients with DED, while the present study was conducted on normal young women. Young women had good muscle function with a small degree of partial blink. Therefore, the blink parameters had no correlation with ocular surface parameters. Moreover, the lack of correlation between LLT and other parameters was in accordance with previous findings, highlighting the difference between the thickness of the lipid layer and its quality.

This study was novel in focusing on the effect of transcutaneous upper blepharoplasty on LLT and blink parameters in young women, while previous studies were mostly carried out on elders and paid no attention to eyeblink parameters and LLT. However, the present study had certain limitations. In the present study, the blink parameters and NITBUT returned to baseline 1 month after the surgery, which was sufficient to observe the effect of upper blepharoplasty on blink parameters. However, the LLT did not return to baseline 1 month after the surgery, which might be related to eyelid edema. In the early stage, the eyelid edema had a dominant role. However, the effect of the removal of the orbicularis muscle on the LLT when edema was eliminated remains unknown. Long-term follow-up of postoperative patients is required to confirm the findings of this study.

## Data Availability Statement

The raw data supporting the conclusions of this article will be made available by the authors, without undue reservation.

## Ethics Statement

The studies involving human participants were reviewed and approved by the Ethics Committee of the Shanghai Ninth People's Hospital. The patients/participants provided their written informed consent to participate in this study.

## Author Contributions

SZ: acquisition of data, drafting the article, final approval of the version to be published, and agreement to be accountable for all aspects of the work. YY: analysis and interpretation of data, drafting the article, final approval of the version to be published, and agreement to be accountable for all aspects of the work. YL: acquisition of data, revising it critically for important intellectual content, final approval of the version to be published, and agreement to be accountable for all aspects of the work. YZ: analysis and interpretation of data, revising it critically for important intellectual content, final approval of the version to be published, and agreement to be accountable for all aspects of the work. YF: substantial contributions to conception and design, revising it critically for important intellectual content, final approval of the version to be published, and agreement to be accountable for all aspects of the work. All authors contributed to the article and approved the submitted version.

## Funding

This research was supported by the National Natural Science Foundation of China (Grant no. 81770888), Shanghai Municipal Education Commission-Gaofeng Clinical Medicine Grant Support (no. 20161421), and Shanghai Ninth People's Hospital Clinical Research Promotion Project (Grant no. JYLJ201904).

## Conflict of Interest

The authors declare that the research was conducted in the absence of any commercial or financial relationships that could be construed as a potential conflict of interest.

## Publisher's Note

All claims expressed in this article are solely those of the authors and do not necessarily represent those of their affiliated organizations, or those of the publisher, the editors and the reviewers. Any product that may be evaluated in this article, or claim that may be made by its manufacturer, is not guaranteed or endorsed by the publisher.
